# Usage status of biologics for the chronic treatment of optic neuritis in neuromyelitis optica spectrum disorders in Japan

**DOI:** 10.1007/s10384-024-01129-4

**Published:** 2024-10-29

**Authors:** Yohei Takahashi, Takeshi Kezuka, Keigo Shikishima, Akiko Yamagami, Hideki Chuman, Makoto Nakamura, Satoshi Ueki, Akiko Kimura, Masato Hashimoto, Sonoko Tatsui, Kimiyo Mashimo, Hitoshi Ishikawa

**Affiliations:** 1 Department of Ophthalmology, Kitasato University School of Medicine, 1-15-1 Kitazato, Minami-ku, Sagamihara-shi, Kanagawa, 252-0373 Japan; 2 Department of Ophthalmology, Tokyo Women’s Medical University, Tokyo, Japan; 3 Department of Ophthalmology, Tokyo Medical University, Tokyo, Japan; 4 Department of Ophthalmology, The Jikei University School of Medicine, Tokyo, Japan; 5 Inouye Eye Hospital, Tokyo, Japan; 6 Department of Ophthalmology, University of Miyazaki, Miyazaki, Japan; 7 Division of Ophthalmology, Department of Surgery, Kobe University Graduate School of Medicine, Hyogo, Japan; 8 Department of Ophthalmology, Niigata University, Niigata, Japan; 9 Department of Ophthalmology, Hyogo Medical University, Hyogo, Japan; 10 Department of Ophthalmology, Nakamura Memorial Hospital, Hokkaido, Japan; 11 Department of Orthoptics and Visual Science, Kitasato University School of Allied Health Sciences, Kanagawa, Japan

**Keywords:** Biologics, Optic neuritis, Oral steroid, Satralizumab, Neuromyelitis optica spectrum disorders

## Abstract

**Purpose:**

To investigate the usage status of biologics for the chronic treatment of optic neuritis including neuromyelitis optica spectrum disorders in Japan.

**Design:**

Multicenter retrospective case series.

**Methods:**

Patients diagnosed with anti-aquaporin 4 antibody (AQP4-Ab) positive optic neuritis and had been initiated on biologics (satralizumab, eculizumab, and inebilizumab) between January 2020 and August 2022 were identified at 30 facilities in Japan. These patients were investigated regarding changes in oral steroid doses, optic neuritis relapse, and adverse events after initiation of biologics.

**Results:**

Eighty-eight patients with AQP4-Ab positive optic neuritis initiated on biologics were included. Satralizumab was the most common biologic used (79 patients), followed by eculizumab (6 patients) and inebilizumab (3 patients). In the satralizumab group, during the observation period (10.0±7.0 months) until February 2023, the oral steroid dose was reduced significantly from 13.8 ± 8.6 mg/day at the time of initiation to 5.3 ± 4.8 mg/day (p < 0.001). No relapse of optic neuritis was observed in 76 of 79 patients (96.2%) after initiation of satralizumab. Furthermore, in 15 patients who succeeded in discontinuing steroids during 8.5 ± 5.8 months after initiation of satralizumab, no relapse of optic neuritis was observed throughout the observation period. Adverse events occurred in 7 patients with satralizumab and 2 patients with eculizumab, but no serious infections were observed.

**Conclusions:**

Satralizumab was the most commonly used biologic for AQP4-Ab positive optic neuritis in Japan. This study demonstrates the efficacy and safety of satralizumab in preventing the relapse of optic neuritis.

## Introduction

Optic neuritis is classified into autoimmune and infectious optic neuritis. Autoimmune optic neuritis, such as optic neuritis associated with neuromyelitis optica spectrum disorders (NMOSD) and anti‒myelin oligodendrocyte glycoprotein antibody (MOG-Ab) ‒associated disorders (MOGAD), is often refractory to treatment and relapses frequently; hence long-term treatment to prevent relapse is important [1]. Since the discovery of anti‒aquaporin-4 antibodies (AQP4-Ab) expressed specifically in NMOSD [2], the clinical picture and related pathological characteristics have become clear [3, 4]. NMOSD may manifest both severe optic neuritis and myelitis. The clinical characteristics of AQP4-Ab positive optic neuritis have been elucidated by an epidemiological study of refractory optic neuritis in Japan [5]. Compared with MOG-Ab positive and double negative (AQP4-Ab negative and MOG-Ab negative) optic neuritis, patients with AQP4-Ab positive optic neuritis are more likely to have poor visual acuity after acute phase treatment. In addition, AQP4-Ab positive optic neuritis is characterized by a higher risk of relapse [6]. Because visual function of AQP4-Ab positive optic neuritis is getting worse at each relapse, effective treatment that prevents relapse is extremely important in the chronic phase of AQP4-Ab positive optic neuritis [7].

Conventionally, oral steroids and immunosuppressants have been the mainstay of preventive treatment for relapse of AQP4-Ab positive NMOSD [8]. However, despite long-term continuation of oral steroids, repeated relapses may occur. Steroid doses are increased following each relapse, and subsequent dose reduction has the risk of inducing further relapses, leading to a situation of steroid dependence, in which some patients are compelled to continue taking considerably high doses of steroids. Long-term treatment with oral steroids is accompanied by the development of various complications including osteoporosis, infections, and glucose intolerance. A previous report proposes the importance of keeping the maintenance dose to the absolute minimum required to avoid adverse events [9].

Considering the above background, recent studies have identified the involvement of various mediators such as complements, interleukin (IL)-6, and B cells in the onset and pathology of AQP4-Ab positive NMOSD [10–13]. Biologic agents possessing actions to control these factors are being introduced as new options of relapse prevention strategy in the chronic phase of NMOSD [14–17]. In Japan, these several types of biologics with different routes of administration and mechanisms of action have become available since 2019, and are expected to be the new options for use in combination with or as an alternative to steroids for preventing optic neuritis relapses. In this study, we conducted a nationwide study in Japan with the aim to clarify the usage status and therapeutic effects of these new drugs.

## Methods

### Participants

This study was a multicenter retrospective case series participated by 30 facilities of a collaborative research group in Japan. The collaborative research group consists of affiliated facilities of ophthalmologists and neurologists with extensive experience in treating optic neuritis and neuromyelitis optica, or affiliated facilities of councilors of the Japanese Neuro-Ophthalmology Society. The participating facilities in this study are distributed nationwide in each of the major regions in Japan, located in 20 of the 47 prefectures in Japan. This research was conducted in compliance with the Declaration of Helsinki, and was reviewed and approved by the ethics committee of each participating facility (representative: Kitasato University Ethics Committee, approval no. B22-115). Since this study was a non-invasive observational study, it was ethically acceptable to obtain verbal informed consent from each patient after explaining the nature of the study.

Patients with NMOSD who started treatment with biological agents between January 1, 2020 and August 31, 2022 for the purpose of preventing relapse in the chronic phase were registered as subjects. In this study, the usage status survey was conducted on three biologics that became available for use under health insurance coverage in Japan during the above study period: satralizumab, eculizumab, and inebilizumab. Satralizumab was administered by subcutaneous injections. Eculizumab and inebilizumab were administered by intravenous infusion.

Exclusion criteria for this study were patients with neuromyelitis optica who manifested only myelitis and no optic neuritis, and patients in whom ophthalmological examinations were not performed before and after administration of the biologics.

Clinical data of the included patients at each facility were retrospectively collected from the medical records, and the investigator at each facility input the information into a questionnaire. Information on clinical characteristics and laboratory test results of the subjects was collected using only existing materials.

### Clinical parameters

The demographic and clinical parameters analyzed included age, sex, biologic used, background of initiation of biologic, main clinical department that administered the biologic (ophthalmology, neurology, others), disease type of NMOSD (optic neuritis alone, or a combination of optic neuritis and myelitis), comorbidities before biologic initiation, and the status of autoantibodies (AQP4-Ab and MOG-Ab). Regarding the evaluation of autoantibodies, AQP4-Ab were determined to be positive when either the enzyme-linked immunosorbent assay (ELISA) method or the cell-based assay (CBA) method was positive. In Japan, only the ELISA method, which has less sensitivity and specificity than the CBA method, is covered by health insurance. Considering that the CBA method is not covered by insurance and, therefore, not available in some facilities, we decided to adopt the autoantibody test results regardless of the measurement method. Since some facilities are unable to use the CBA method, only those cases that had MOG-Ab measured and showed a positive reaction were determined to be seropositive for MOG-Ab. In addition, we investigated whether immunosuppressive drugs were used as concomitant treatment other than steroids, and if used, the type of drug and the status of continuation after biologic initiation were also examined.

### Efficacy of biologics

The efficacy outcomes of initiating biologics for optic neuritis included the change in oral steroid dose and the change in number of optic neuritis attacks before and after initiation of each biologic. The number of patients initially receiving eculizumab or inebilizumab was small, and so specific analysis of efficacy was performed only in the satralizumab group.

#### Dose reduction and discontinuation of steroids

In this analysis, patients who discontinued or switched the biologic due to occurrence of adverse events, and patients in whom steroid administration was irregular after initiation of biologic were excluded.

For patients who were initiated with satralizumab (the largest number of patients among the three biologics), during the average observed period from the start of the biologic to February 28, 2023, the average oral steroid dose (mg) at biologic initiation, and the average final steroid dose (mg) within the observed period were calculated. The dose of oral steroids at initiation and the last dose within the observation period were compared statistically. In addition, the average oral steroid dose at 6 months and 12 months in patients who continued oral steroids for 12 months or longer after initiation of satralizumab were calculated and compared statistically with the dose at its initiation.

For patients who were using oral steroids at initiation of a biologic and later discontinued the steroids during the observation period, the number of patients, the dose of oral steroids at initiation of biologic, and the period from initiation of biologic to discontinuation of steroids were investigated.

#### Number of optic neuritis attacks

In this analysis, the following were investigated: observed period from the start of biologic to February 28, 2023, the number of optic neuritis attacks before initiation of biologic, and the number of optic neuritis attacks during the observed period after initiation of biologic. Regarding attacks before initiation of biologic, the total number of attacks from the onset of optic neuritis to the time of biologic initiation was investigated.

For patients who were initiated with satralizumab, the most common biologic used, the average number of attacks before initiation and that after initiation were compared. For patients who developed NMOSD more than 1 year before initiation of satralizumab and were followed up for more than 1 year after initiation of satralizumab, the number of optic neuritis attacks within both 1 year before and after initiation of satralizumab were investigated and compared as annual relapse rate (ARR). Moreover, the proportion of patients with no optic neuritis attacks during the observable period after initiation was evaluated.

For patients who had relapse of optic neuritis after initiation of satralizumab, the following were investigated: age, history of myelitis before initiation, number of optic neuritis attacks before and after initiation, first relapse after initiation in months, acute treatment for relapse, dose of oral steroids (at initiation of biologic, at relapse, and after acute treatment for relapse), concomitant use of immunosuppressants, and change of biologic after relapse.

### Safety of biologics

For safety evaluation of the biologics, the status of occurrence of any adverse event and the status of occurrence of specific adverse events were investigated. The specific adverse events listed were those with relatively high frequencies selected from the adverse events shown in the package inserts of the three biologics, such as infections, hepatic impairment, decreased blood cell counts, anaphylaxis, and infusion reaction. Occurrence of other adverse events, if any, was entered in the form of free description. Information on continuing or discontinuing the biologic after occurrence of adverse event and the measures taken during occurrence of adverse event were also collected.

### Statistical analyses

In evaluating the efficacy of satralizumab, the biologic used by the largest number of subjects, statistical analysis was conducted on the change in oral steroid dose and the annualized relapse rate of optic neuritis before and after initiation of the biologic. Paired Student's t-tests were used for the following comparisons: the initial starting dose vs. final dose for patients who continued satralizumab within the study period, initial starting dose vs. dose at 6 months after initiation and initial starting dose vs. dose at 12 months after initiation in patients who continued oral steroids for more than 12 months after initiation of satralizumab, and the annualized relapse rate within 1 year before and after initiation of satralizumab. In the repeated measure analysis of variance, we calculated the required sample size with an effect size f of 0.25 (middle size), α error of 0.05, and power of 80%; the required sample size was calculated to be 28 cases. The test results were defined as statistically significant if the p value was less than 0.05. All analyses were performed using the statistical program R version 4.1.1 (R Foundation for Statistical Computing).

## Results

### Participant characteristics

A total of 88 patients with NMOSD treated initially with one of the three biologics were identified. The most commonly used biologic at the time of initiation was satralizumab (79 patients), followed by eculizumab (6 patients), and inebilizumab (3 patients) (Table 1). All 88 patients were seropositive for AQP4-Ab and only one patient was seropositive for both AQP4-Ab and MOG-Ab. The most common reason for initiating a biologic was to avoid the risk of blindness due to optic neuritis (51 patients), followed by dose reduction or discontinuation of oral steroids (38 patients). The primary clinical department that administered the biologic at each facility was ophthalmology in 23 patients (8 ophthalmology facilities), neurology in 62 patients (17 ophthalmology facilities and 3 neurology facilities), and pediatrics in 3 patients (3 ophthalmology facilities) (Table 1).

**Table 1:** Clinical characteristics of NMOSD patients with optic neuritis treated with biologics (Satralizumab, Eculizumab and Inebilizumab)

	Satralizumab (n = 79)	Eculizumab (n = 6)	Inebilizumab (n = 3)
Age, years, mean ± SD (range)	51.9 ± 15.5 (11–87)	52.0 ± 16.6 (19–66)	69.7 ± 11.2 (57–78)
Sex, female, n (%)	73 (92.4)	5 (83.3)	1 (33.3)
Main clinical department, *n* (%)			
Ophthalmology	22 (27.8)	0	1 (33.3)
Neurology	54 (68.4)	6 (100.0)	2 (66.7)
Pediatrics	3 (3.8)	0	0
NMOSD type, n (%)			
ON only	35 (44.3)	2 (33.3)	1 (33.3)
ON with myelitis	44 (55.7)	4 (66.7)	2 (66.7)
Comorbidities, n			
Diabetes	6	3	0
Hepatic impairment	8	1	0
Collagen disease	4	2	0
Others	30	2	1
ISD, *n* (%)	31 (39.2)	4 (66.7)	0
AZP	21	3	0
TAC	8	1	0
CYA	3	0	0
Discontinued after biologic initiation	10	2	0

*NMOSD* neuromyelitis optica spectrum disorders, *SD* standard deviation, *ON* optic neuritis, *ISD* immunosuppressive drugs, *AZ* azathioprine, *TAC* tacrolimus, *CYA* cyclosporin.

Regarding the disease type of AQP4-Ab positive NMOSD, 38 patients manifested optic neuritis alone, and 50 patients had myelitis concurrent with optic neuritis (Table 1). In all three biologic groups, there were more patients with concurrent optic neuritis and myelitis than those with optic neuritis alone. Thirty-five patients (31 patients in satralizumab group, 4 patients in eculizumab group, and 0 patients in the inebilizumab group) were taking concomitant immunosuppressants at the time of initiation of the biologic (Table 1). Azathioprine was the most commonly used immunosuppressant. After initiation of biologic, 12 patients (10 patients in satralizumab group and 2 patients in eculizumab group) managed to discontinue the immunosuppressants.

### Efficacy of biologics

#### Dose reduction and discontinuation of steroids

For the study of dose changes and discontinuation of steroids, 73 patients in the satralizumab group (after excluding 3 patients who discontinued treatment due to adverse events and 3 who took steroids irregularly after initiation of the biologic), 5 patients in the eculizumab group (after excluding 1 who discontinued treatment due to adverse event), and all 3 patients in the inebilizumab group were included.

**Fig. 1:**
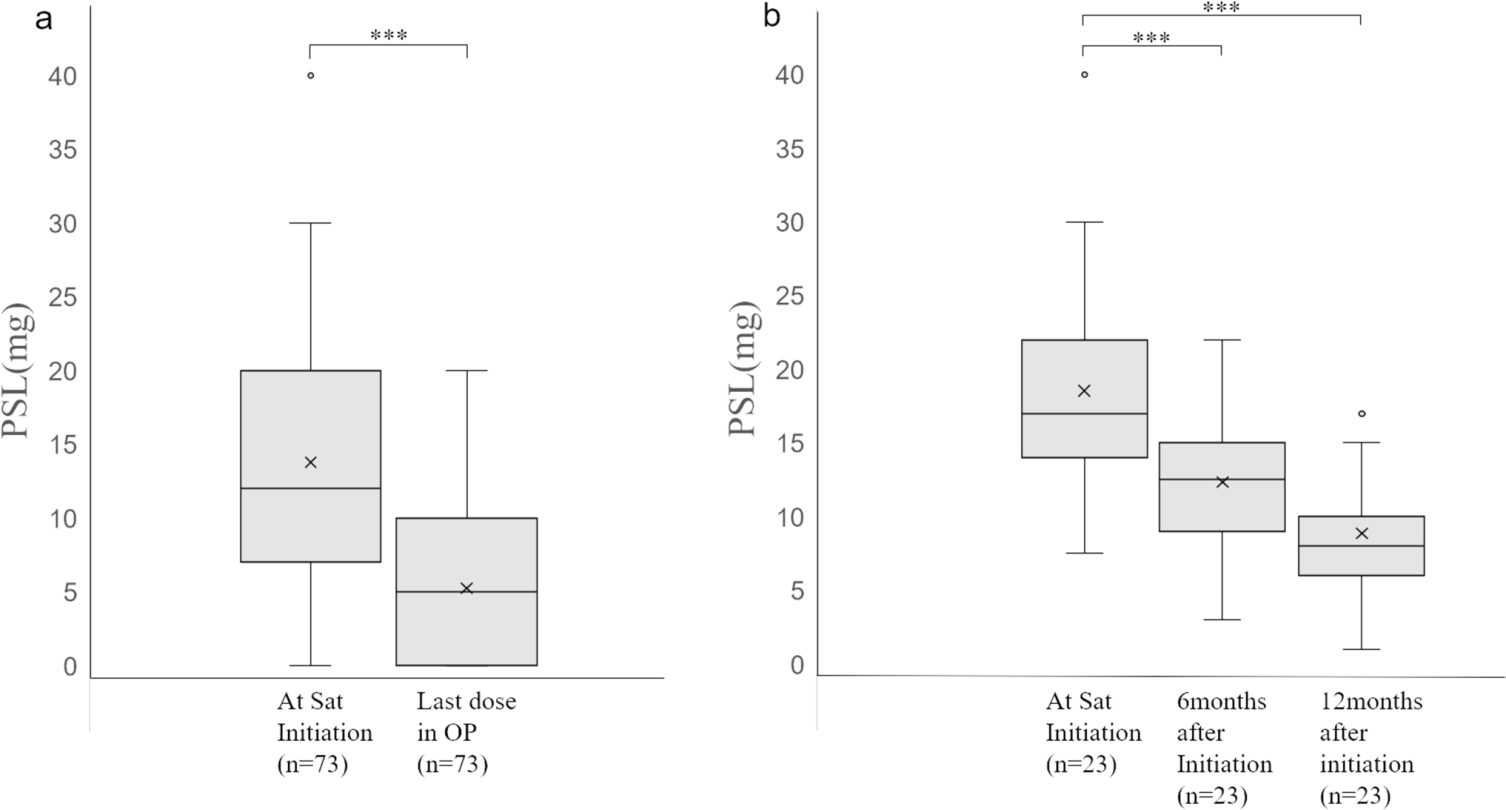
Comparison of oral steroid doses at initiation and after initiation of satralizumab. **a** Oral steroid doses in 73 patients with optic neuritis at initiation of satralizumab and at the last dose within the observable period after initiation. Compared to the dose at satralizumab initiation of 13.8 ± 8.6 mg/day, the final dose within the observation period decreased significantly to 5.3 ± 4.8 mg/day (***p < 0.001). b: Oral steroid doses at initiation of satralizumab, at 6 months after initiation, and at 12 months after initiation in 23 patients who were observable for 12 months or longer after initiation. The dose at 6 months after initiation (12.3 ± 4.6 mg/day) and that at 12 months after initiation (8.9 ± 4.0 mg/day) decreased significantly compared to the dose at satralizumab initiation (***p < 0.001 for both). *PSL* prednisolone, *Sat* satralizumab, *OP* observation period.

The changes in oral steroid dose from initiation of satralizumab to the last dose of the observation period were analyzed (Table 2). The 73 patients in the satralizumab group achieved dose reduction from an average dose of 13.8 ± 8.6 (0‒40) mg/day at initiation to 5.3 ± 4.8 (0‒20) mg/day at the last dose within the observation period (10.0 ± 7.0 months), with a significant difference (p < 0.001) (Fig. 1a). In the 23 patients who were using oral steroids for more than 12 months after initiation of satralizumab, compared to the average dose of 18.5 ± 7.7 (7.5‒40) mg/day when satralizumab was initiated, the dose at 6 months after initiation was 12.3 ± 4.6 (3‒22) mg/day, and the dose at 12 months was 8.9 ± 4.0 (1‒17) mg/day, with significant dose reduction in both groups (p < 0.001, for both) (Fig. 1b).

**Table 2:** Dose change or discontinuation of steroids from initiation of satralizumab for NMOSD (n=73)

	Satralizumab (n = 73)
Observation period, months, mean ± SD (range)	10.0 ± 7.0 (1–25)
PSL dose, mg, mean ± SD (range)	
At initiation of biologic	13.8 ± 8.6 (0–40)
Final dose of observation period	5.3 ± 4.7 (0–20)
Cases of PSL discontinuation, n	15
Dose at initiation of biologic, mg, mean ± SD (range)	11.6 ± 8.8 (1–30)
Duration until PSL discontinuation, months, mean ± SD (range)	8.5 ± 5.8 (1–21)

*NMOSD* neuromyelitis optica spectrum disorders, *SD* standard deviation, *PSL* prednisolone

In the eculizumab group (5 patients), steroid dose at initiation was 10.1 ± 5.4 (5‒18) mg/day and the last dose within the observation period (13.2 ± 7.5 months) was 3.4 ± 2.9 (0‒8) mg/day. In the inebilizumab group (3 patients), steroid dose at initiation was 11.0 ± 2.9 (8‒15) mg/day and the last dose within the observation period (4.3 ± 2.6 months) was 4.7 ± 3.7 (0‒9) mg/day.

Among the patients who were using oral steroids at initiation of biologic, 17 patients were able to discontinue oral steroids during the observation period. These patients comprised 15 patients in the satralizumab group (Table 2), and 1 patient each in the eculizumab group and inebilizumab group. In the 15 patients who discontinued steroids after initiation of satralizumab, the oral steroid dose at initiation of satralizumab was 11.6 ± 8.8 (1‒30) mg, and the duration from satralizumab initiation to steroid discontinuation was 8.5 ± 5.8 (1‒21) months (Table 2), with no relapse in all 15 patients during the observation period after steroid discontinuation. There was also no relapse during the observation period in the 4 patients not using steroids at initiation of satralizumab. One patient in the eculizumab group was using 5 mg of steroids at the time of initiation and required 21 months to discontinue steroids, while 1 patient in the inebilizumab group using 8 mg of steroids at initiation required 3 months until discontinuation.

#### Number of optic neuritis attacks

In the satralizumab group (79 patients), 39 patients had one optic neuritis attack, 20 patients had two relapses, and 20 patients had three or more attacks (maximum 16) before initiation of a biologic; whereas 96.2% (76/79) of the patients who were treated initially with satralizumab were free of relapse and only 3 patients had relapse during the observation period after initiating biologic (no relapse in 76 patients, one relapse in 2 patients, and two relapses in 1 patient) (Table 3). In all 79 patients, the average number of optic neuritis attacks was 2.43 ± 2.76 before initiation of satralizumab, and the number of attacks after initiation of satralizumab was 0.05 ± 0.27, within the observation period (10.0 ± 7.1 months).

**Table 3:** Number of attacks of optic neuritis before and after initiation of satralizumab for NMOSD (n=79)

	Satralizumab (n = 79)
Observation period, months, mean ± SD (range)	10.0 ± 7.1 (1–25)
No. of attacks before biologic initiation	
One, n	39
Two, n	20
≥ Three, n	20
No attack after biologic initiation, n (%)	76 (96.2)

*NMOSD* neuromyelitis optica spectrum disorders, *SD* standard deviation

Of the 79 patients with satralizumab, twenty-nine patients were observed for more than 1 year before and after initiation of satralizumab. Among these 29 patients, 12 patients had 12 optic neuritis relapses within one year before initiation of satralizumab, and 2 patients had 3 relapses within one year after initiation of satralizumab. The annualized relapse rate (ARR) [95% confidence interval: CI] was 0.41 [0.22–0.60] per person within 1 year before initiation of satralizumab and 0.10 [− 0.05–0.26] within 1 year after initiation of satralizumab, showing a significant decrease after initiation of satralizumab compared with before initiation of satralizumab (p=0.009).

Of the 3 patients with relapse of optic neuritis after initiation of satralizumab, 1 patient (Case 1) had optic neuritis alone before initiation of satralizumab, and the other 2 patients (Case 2 and 3) had a history of developing optic neuritis and myelitis at different times (Table 4). Satralizumab was continued without switching to another biologic in one patient (Case 1) with optic neuritis only, while satralizumab was switched to eculizumab in two patients (Case 2 and 3) who had optic neuritis and myelitis (Table 4). Case 1, a 13-year-old child, had no history of myelitis before initiation of satralizumab, but had frequent relapses of optic neuritis, including 5 attacks. From the time satralizumab was initiated, PSL 3 mg/day was administered in combination with tacrolimus and cyclosporine, and none of the treatments were reduced or discontinued, but optic neuritis recurred 7 months after the initiation of satralizumab. At the time of relapse, steroid pulse therapy was performed, followed by an increase in PSL to 10 mg/day, and satralizumab was continued, but the patient subsequently experienced a second relapse of optic neuritis. Both cases, Case 2 and 3, had a history of myelitis at a time separate from the optic neuritis attack before initiation of satralizumab. No immunosuppressants were used at the time of initiation, and the oral dose of PSL was continued unchanged from the time of initiation (Case 2: 7.5 mg/day, Case 3: 10 mg/day), but optic neuritis recurred within 6 months of initiation. After steroid pulse therapy at the time of relapse, Case 2 was kept at PSL: 7.5 mg/day, and Case 3 was increased to PSL: 15 mg/day, and satralizumab was changed to eculizumab.

**Table 4:** Clinical data of patients with relapse of optic neuritis after initiation of satralizumab for NMOSD (n=3)

	Case 1	Case 2	Case 3
Age, years,	13	54	44
History of myelitis before initiation	No	Yes	Yes
No. of ON relapses			
before initiation	5	2	1
after initiation	2	1	1
Months to relapse after initiation	7	5	4
Acute treatment for relapse	SP	SP	SP
PSL dose, mg			
at initiation of biologic	3	7.5	10
at relapse	3	7.5	10
after acute treatment for relapse	10	7.5	15
Immunosuppressive drugs	TAC+CYA	None	None
Change of biologic after relapse	No change	Switch (eculizumab)	Switch (eculizumab)

*NMOSD* neuromyelitis optica spectrum disorders, *ON* optic neuritis, *SP* steroid pulse, *PSL* prednisolone, *TAC* tacrolimus, *CYA* cyclosporin.

In the eculizumab group (6 patients, observation period was 11.3 ± 8.0 months) and inebilizumab group (3 patients, observation period was 4.3 ± 2.6 months), all patients experienced one or two attacks of optic neuritis before initiation of biologic, and none of the patients had relapse during the observation period after initiating biologic.

### Adverse events of biologics

Adverse events were observed after initiation of biologic in 7 patients in the satralizumab group and 2 patients in the eculizumab group. The adverse events in the satralizumab group are summarized in Table 5.

**Table 5:** Summary of adverse events after initiation of satralizumab for NMOSD (n=7)

AEs	Age (years)	Measure for biologic	Measure and outcome
Infection	26	Continued	Admitted, improved with observation
Cytopenia	53	Continued	Prolonged interval of satralizumab dosing
Cytopenia	58	Continued	Modified other co-administered drugs
Headache	40	Continued	Improved with observation
Hepatic impairment	29	Discontinued (switched)	Switched to inebilizumab
Anal cancer	40	Discontinued	Treatment for malignant tumor
Ovarian cancer	47	Discontinued	Treatment for malignant tumor

*NMOSD* neuromyelitis optica spectrum disorders, *AEs* adverse events.

Of the 7 patients with adverse events in the satralizumab group, 4 patients continued satralizumab, and 3 patients either discontinued biologic treatment or switched to another biologic. Adverse events in the 4 patients who continued satralizumab comprised suspected infection, decreased blood cell counts, and headache, but none of them were serious and all improved with conservative observation; as a result, satralizumab was continued without switching to another biologic. Among the 3 patients who discontinued satralizumab, hepatic impairment developed in 1 patient and satralizumab was switched to inebilizumab, while malignant tumors were detected in 2 patients after initiation, and satralizumab was discontinued in both patients, although the causal relationship with the biologic was unclear. Of these two cases in which malignant tumors were detected, one with anal cancer was not receiving concomitant immunosuppressants, and the other with ovarian cancer was receiving azathioprine in combination with the biologic agent at the time of initiation.

In the eculizumab group, 1 patient suspected of having an infection improved with antibiotic treatment and eculizumab was continued. Another patient with exacerbated concurrent systemic lupus erythematosus discontinued eculizumab 2 months after initiation, and oral steroid dose was increased from 10 to 20 mg.

## Discussion

In the present study, we investigated the usage status of three biologic agents in Japan: satralizumab, eculizumab and inebilizumab, in patients with AQP4-Ab positive NMOSD, focusing on those with newly diagnosed or relapsed optic neuritis. The indication of these biologics is limited to AQP4-Ab positive cases. In other words, the effectiveness of these agents against AQP4-Ab negative cases has not been established. All the cases analyzed in this study were seropositive for AQP4-Ab, and only one case was seropositive for both AQP4-Ab and MOG-Ab.

In our study, satralizumab was the most commonly used agent among the three biologics. It is reported that, in the United States, between November 2022 and July 2023, rituximab (53%) was the most common treatment for 127 cases of NMOSD aged 18-70 years old, followed by satralizumab (8%), eculizumab (7%), and inebilizumab (4%) [18].

The difference in usage status of the biologics in our study is probably related to the administration methods of the biologics. Because satralizumab can be self-injected once the patient is familiar with the subcutaneous injection procedure, it may be easier to start satralizumab treatment. In this study, the departments participating in the survey varied by facility, resulting in bias; however, even at facilities where the department in charge was ophthalmology, the biologics were often administered by neurologists. Because of the advantages of the subcutaneous injection of satralizumab described above, it is possible that its use in ophthalmology may increase in the future.

### Efficacy of satralizumab in achieving dose reduction or discontinuation of immunosuppressants and steroids

After completion of acute phase treatment for NMOSD, relapse prevention treatment using conventional oral steroid monotherapy is not considered cost-effective considering the steroid-induced adverse events [19], as well as that the relapse-free rate of only 46.5% over 10 years is not necessarily high [9]. In addition, when using steroids alone to prevent relapse of NMOSD, the period of 12 months from the initial onset or relapse is considered to be a high-risk period for “cluster attacks”. Aggressive treatment with relapse prevention therapies during this period is deemed necessary [20]. The initiation of immunosuppressive drugs or biologic agents to replace steroids is becoming accepted as the first-line treatment for relapse prevention. Regarding immunosuppressive drugs, the antimetabolite azathioprine is reported to be frequently used and effective [8]. In the present study, the majority of the patients used azathioprine concomitantly at the initiation of satralizumab or eculizumab, and 12 of 36 patients who had been co-administered immunosuppressants were able to discontinue the immunosuppressants after starting the biologic. Since these immunosuppressive drugs have unique adverse events, it is necessary to consider the differential use or combined use with biologics for individual patients.

When oral steroids are used in combination with immunosuppressive drugs or biologic agents, it is recommended to start with a low to medium dose that is minimally required and start tapering after several to six months to avoid adverse events [8]. In the case of using steroids alone, gradual tapering (reduce the daily dose by 1 mg every 1–2 months, with the daily dose reduced to approximately 15 mg at 1 year after starting) is considered relatively safe [9]. In the case of combined use, more aggressive tapering (daily dose reduced to 10 mg or lower at 1 year after starting) is possible. Regarding steroid tapering after the initiation of biologics, a report shows that in patients participating in the SAkuraSky who were administered satralizumab combined with steroids and immunosuppressants, reducing the dose of oral steroids was possible without worsening the underlying disease [21]. In the SAkuraSky study, the steroid dose was tapered from a median of 10.0 mg at initiation to 2.75 mg at the end of study. Two patients had a total of three relapses during steroid tapering, but no relapse was observed in any of the three patients who succeeded to discontinue steroids entirely. A publication reports 3 cases of NMOSD that succeeded to taper steroid doses to less than 3 mg/day after starting satralizumab [22]. From 8 weeks after initiation of satralizumab, the daily dose of oral steroids was reduced by 1 mg every 4 weeks. In that study, steroid tapering was started 8 weeks after initiating satralizumab based on the evidence that serum soluble IL-6 receptor level plateaus approximately 8 weeks after starting satralizumab, so that the effects of satralizumab may be expected to stabilize from 8 weeks after initiation. Recently, it is reported that the number of patients on 0 mg/day dose of oral steroids without relapse increased over time under continuous satrarizumab prescription [23].

In the present study, many patients who continued satralizumab without relapse or adverse event during the observation period were able to reduce the steroid dose to less than 10 mg/day between 6 and 12 months after starting satralizumab. In most patients, a given steroid dose was taken for 4 weeks and then reduced by 1 mg/day on the day of satralizumab administration (injected once every four weeks), and this step-wise steroid dose reduction was repeated at intervals of 4 weeks. Steroid tapering after initiation of satralizumab was possible in our study at a rate almost equivalent to the study reported in 2023 [22]. In addition, no relapse was observed in patients who did not use steroids at the initiation of biologic and in patients who were able to discontinue steroids after the biologic was started; meanwhile none of the patients had relapse because the steroid tapering speed was too rapid. However, since the relapse prevention effect may be insufficient soon after the biologic is initiated, it may be appropriate to start tapering after a certain time of biologic treatment, or to change the tapering speed at around 8 weeks after tapering is started. Although it is unclear what cases are prone to relapse after initiating biologic, in patients with known risks of relapse such as frequent relapses before starting a biologic, steroid tapering has to be done gradually and cautiously by bearing in mind the appropriate speed for individual patients.

The advantage of biologics is that successful tapering or discontinuation of steroids would reduce the risk of various adverse events caused by long-term use of oral steroids. In the present study, the majority of patients were on moderate doses of oral steroids when biologics were started. However, if it is possible to start biologics immediately after an acute phase treatment, the disease can be managed by biologics alone without using oral steroids. Biologics are especially recommended when functional impairment interferes markedly with activities of daily living even at the initial onset, when poor response to acute treatment leads to residual irreversible disability, when aggressive prevention of relapse is desirable, or when use of steroids is a concern due to the risk of comorbidity. It is necessary to consider on an individual basis whether the advantages of choosing biologic agents outweigh the disadvantages of steroid use. Since both biologic agents and immunosuppressive drugs have adverse events, it would be desirable to select the priority drugs that provide adequate control for individual patients by weighing the efficacy of relapse prevention against the risk of adverse events for each drug.

### Prevention of optic neuritis relapse by initiating satralizumab

The efficacy of satralizumab has been evaluated in two trials: SAkuraSky and SAkuraStar. The subjects in SAkuraSky were treated with steroids and immunosuppressants, while those in SAkuraStar were treated with satralizumab monotherapy without immunosuppressants. The relapse rate during the double-blind study period was 11% (SAkuraSky) and 22% (SAkuraStar) of patients treated with satralizumab seropositive for AQP4-Ab [14, 15]. In both trials, satralizumab showed a significant relapse prevention effect compared to placebo. The results of long-term evaluation of both trials were reported in 2023 [24]. Regarding long-term efficacy, the relapse rate was 24% (SAkuraSky) and 27% (SAkuraStar) in AQP4-Ab positive patients.

In the present study, the relapse rate of patients with AQP4-Ab positive optic neuritis treated with satralizumab was as low as in previous reports, and the relapse rate was significantly reduced one year before and after the initiation of satralizumab, supporting the finding that satralizumab has a high relapse prevention effect. Nakashima et al. report that 95.4% of patients with NMOSD were relapse-free in a median duration of satralizumab exprosure of 197.0 days [23]. A difference from previous studies is that the present study selected patients with optic neuritis alone or with optic neuritis and myelitis, and did not include patients with myelitis alone. We speculate that the effect of satralizumab in preventing relapse may be better against optic neuritis than against myelitis. However, since the observation period in this study (less than two years) was shorter compared with previous trials, it is possible that the number of relapses may increase if the follow-up period is further extended.

In our study, only a small number of patients had relapse while using the biologic, and the measures taken for the relapse differed among patients. Several patients continued treatment without changing the biologic after taking measures such as increasing the dose of steroids. However, if the physician determines that the biologic has inadequate or no relapse prevention effect, switching to another biologic agent may be advisable.

### Safety of satralizumab

Satralizumab is a monoclonal antibody against IL-6 receptor involved in the pathology of NMOSD. IL-6 is a cytokine involved in inflammatory reactions, and the IL-6 inhibitory effect of satralizumab suppresses fever symptoms and increase in blood level of C-reactive protein, which poses a risk of delaying the detection of infection. For this reason, contraindications for using satralizumab are concurrent serious infection and active tuberculosis, and infections are the complication that require special attention after initiation of the biologic. In particular, there is a risk of exacerbation of urinary tract infection and respiratory infection, therefore, caution is required. The safety of satralizumab has been evaluated in SAkuraSky [14] and SAkuraStar [15]. Adverse events occurred in 41.5% of patients in SAkuraSky and in 34.9% of patients in SAkuraStar. A report of long-term study of both SAkuraSky and SAkuraStar shows that long-term use of satralizumab did not increase the frequency of serious adverse events or infections compared to placebo, and there were no deaths and no reports of anaphylactic shock [25].

In the present study, there were only 7 adverse events in the satralizumab group, 4 of which were mild, and there were no serious infections necessitating discontinuation of the biologic or administration of antibiotics. In addition, two cases of malignant tumor were detected after satralizumab was initiated, resulting in discontinuation of biologic treatment. Although the causal relationship between anti-IL-6 agents and the risk of developing malignant tumors is unknown, one report shows that 10–20% of patients with NMOSD had a pre-onset history of malignant tumor [26]. Moreover, among immunosuppressants, azathioprine is known to be associated with a risk of malignant tumors, and one patient in this study was also administered azathioprine concomitantly. In light of the above, it may be necessary to conduct a thorough evaluation of the patient’s general conditions including screening for malignant tumors before initiating biologic agents in NMOSD patients.

In our study, the methods of managing adverse events that occurred during use of biologics differed among patients. For minor adverse events that improved with observation, the biologic was continued without switching. However, especially when continuation of the biologic has the risk of causing irreversible damage, such as serious infection or malignant tumor, it may be advisable to switch to another type of biologic or to discontinue using biologics altogether. Even though the incidence of serious adverse events that include infections and infusion reactions is not high, it may be necessary at the initiation of biologics to consider carefully the measures to be taken when these adverse events occur.

### Eculizumab and inebilizumab

For eculizumab and inebilizumab, the efficacy in preventing the relapse of NMOSD and the safety are reported in a trial and the extended long-term analysis [16, 17, 27, 28]. Regarding comparison of the relapse prevention effects of different biologics, a meta-analysis comparing eculizumab, satralizumab, and inebilizumab shows that eculizumab tended to have a lower risk of relapse compared to the other biologics, regardless of whether the patients were treated with a biologic alone or with biologic combined with immunosuppressive therapy [29].

In the present study, no relapse and no serious adverse events were observed in either the eculizumab group or the inebilizumab group. However, the number of patients initially receiving eculizumab or inebilizumab was small, and so it was not possible to conduct a statistical analysis on dose reduction of steroids and the relapse rates before and after initiation of the biologic. Moreover, due to the short observation period, the results obtained do not reflect long-term outcomes. Further analysis of a larger number of patients for eculizumab and inebilizumab in longer observation period is required to evaluate the efficacy and safety of these two biologics.

### Other biologics

There exist some novel biologics, rituximab and ravulizumab; these two drugs were not included in this study because they had not been approved by the time of the retrospective survey period. Rituximab has been shown to be effective in suppressing relapses in NMOSD [30, 31], it is also used clinically for autoimmune diseases other than NMOSD. The administration interval during the maintenance phase is long, and the drug price is lower than other biological agents. For these reasons, it is possible that its use will increase. In addition, ravulizumab shows the same mechanism of action and adverse events as eculizumab [32], and the administration interval during the maintenance phase is longer than eculizumab at every 8 weeks, so the number of cases in which its introduction can be considered may increase by reducing the frequency of administration. It will be necessary to consider the practical value of all biological agents, including these new agents, in the future.

### Study limitations

A limitation of the present study is that this retrospective observational study focusing on optic neuritis using only existing materials does not allow comparison of the efficacy of each biologic agent with controls. In evaluating the efficacy of satralizumab, the mean follow-up period was relatively short, 10.0 months, and we may not have evaluated its long-term efficacy. In addition, because the number of patients who continued oral steroids for 12 months or more was less than the required minimum sample size, it may not be possible to detect a sufficient difference when comparing the results of oral steroid doses after 6 and 12 months. Because eculizumab and inebilizumab were used in markedly fewer patients than satralizumab, it was not possible to compare the effectiveness of the three biologic agents, or to determine the indications for different patients based on the characteristics of the biologics. Further analysis of a larger number of patients for each biologic agent is required to verify the present findings.

The present study demonstrates that initiation of the biologic agents satralizumab in patients with NMOSD demonstrates the possibility of reducing the dose of or discontinuing steroids after initiation, and was highly effective in preventing relapse of AQP4-Ab positive optic neuritis. In NMOSD, satralizumab is believed to be more effective in preventing relapse of optic neuritis than of myelitis. Biologics may have major advantages as a novel chronic phase treatment for refractory optic neuritis, such as superior relapse prevention effect and avoidance of adverse events associated with steroid use. Further studies with a larger number of patients observed over a longer period of time is expected to define measures to address infections and other adverse events specific for biologics and to explore the possibility of initiating biologics immediately after completion of acute phase treatment.

## References

[1] Petzold A, Fraser CL, Abegg M, Alroughani R, Alshowaeir D, Alvarenga R, et al. Diagnosis and classification of optic neuritis. Lancet Neurol. 2022;21:1120–34. 36179757 10.1016/S1474-4422(22)00200-9

[2] Lennon VA, Wingerchuk DM, Kryzer TJ, Pittock SJ, Lucchinetti CF, Fujihara K, et al. A serum autoantibody marker of neuromyelitis optica: distinction from multiple sclerosis. Lancet. 2004;364:2106–12. 15589308 10.1016/S0140-6736(04)17551-X

[3] Wingerchuk DM, Banwell B, Bennett JL, Cabre P, Carroll W, Chitnis T, et al. International consensus diagnostic criteria for neuromyelitis optica spectrum disorders. Neurology. 2015;85:177–89. 26092914 10.1212/WNL.0000000000001729 PMC4515040

[4] Trebst C, Jarius S, Berthele A, Paul F, Schippling S, Wildemann B, et al. Update on the diagnosis and treatment of neuromyelitis optica: Recommendations of the Neuromyelitis Optica Study Group (NEMOS). J Neurol. 2014;261:1–16. 24272588 10.1007/s00415-013-7169-7 PMC3895189

[5] Ishikawa H, Kezuka T, Shikisima K, Yamagami A, Hiraoka M, Chuman H, et al. Epidemiologic and clinical characteristics of optic neuritis in Japan. Ophthalmology. 2019;126:1385–98. 31196727 10.1016/j.ophtha.2019.04.042

[6] Contentti C, Virgiliis M, Hryb JP, Gomez A, Morales S, Celso J, et al. Aquaporin-4 Serostatus and Visual Outcomes in Clinically Isolated Acute Optic Neuritis. J Neuro-Ophthalmol. 2019;39:165–9. 10.1097/WNO.0000000000000668 30004999

[7] Nakazawa M, Ishikawa H, Sakamoto T. Current understanding of the epidemiologic and clinical characteristics of optic neuritis. Jpn J Ophthalmol. 2021;65:439–47. 34021411 10.1007/s10384-021-00840-w

[8] Fujihara K. Neuromyelitis optica spectrum disorders: still evolving and broadening. Curr Opin Neurol. 2019;32:385–94. 30893099 10.1097/WCO.0000000000000694 PMC6522202

[9] Takai Y, Kuroda H, Misu T, Akaishi T, Nakashima I, Takahashi T, et al. Optimal management of neuromyelitis optica spectrum disorder with aquaporin-4 antibody by oral prednisolone maintenance therapy. Mult Scler Relat Disord. 2021;49: 102750. 33524925 10.1016/j.msard.2021.102750

[10] Ratelade J, Asavapanumas N, Rithie AM, Wemlinger S, Bennet JL, Verkman AS. Involvement of antibody-dependent cell-mediated cytotoxicity in inflammatory demyelination in a mouse model of neuromyelitis optica. Acta Neuropathol. 2013;126:699–709. 23995423 10.1007/s00401-013-1172-z PMC3890328

[11] Ratelade J, Zhang H, Saadoun S, Bennett JL, Papadopoulos MC, Verkman AS. Neuromyelitis optica IgG and natural killer cells produce NMO lesions in mice without myelin loss. Acta Neuropathol. 2012;123:861–72. 22526022 10.1007/s00401-012-0986-4 PMC3581313

[12] Chihara N, Aranami T, Sato W, Miyazaki Y, Miyake S, Okamoto T, et al. Interleukin 6 signaling promotes anti-aquaporin 4 autoantibody production from plasmablasts in neuromyelitis optica. Proc Natl Acad Sci USA. 2011;108:3701–6. 21321193 10.1073/pnas.1017385108 PMC3048150

[13] Fujihara K, Bennet JL, Jerome S, Haramura M, Kleiter I, Weinshenker BG, et al. Interleukin-6 in neuromyelitis optica spectrum disorder pathophysiology. Neurol Neuroimmunol Neuroinflamm. 2020;7: e841. 32820020 10.1212/NXI.0000000000000841 PMC7455314

[14] Yamamura T, Kleiter I, Fujihara K, Palace J, Greenberg BM, Zakrzewska-Pniewska B, et al. Trial of satralizumab in neuromyelitis optica spectrum disorder. N Engl J Med. 2019;381:2114–224. 31774956 10.1056/NEJMoa1901747

[15] Traboulsee A, Greenberg BM, Bennett JL, Szczechowski L, Fox E, Shkrobot S, et al. Safety and efficacy of satralizumab monotherapy in neuromyelitis optica spectrum disorder: a randomised, double blind, multicentre, placebo-controlled phase 3 trial. Lancet Neurol. 2020;19:402–12. 32333898 10.1016/S1474-4422(20)30078-8 PMC7935419

[16] Pittock SJ, Berthele A, Fujihara K, Kim HJ, Levy M, Palace J, et al. (2019) Eculizumab in aquaporin-4–positive neuromyelitis optica spectrum disorder. N Engl J Med. 2019;381:614–25. 31050279 10.1056/NEJMoa1900866

[17] Cree BAC, Bennet JL, Kim HJ, Weinshenker BG, Pittock SJ, Wingerchuk DM, et al. Inebilizumab for the treatment of neuromyelitis optica spectrum disorder (N-MOmentum): a double-blind, randomised placebo-controlled phase 2/3 trial. Lancet. 2019;394:1352–63. 31495497 10.1016/S0140-6736(19)31817-3

[18] Hjerthen IG, Trápaga Hacker C, Meador W, Obeidat AZ, Horta L, Mateen FJ. Impact of neuromyelitis optica spectrum disorder on employment and income in the United States. Ann Clin Transl Neurol. 2024;11:1011–20. 38374778 10.1002/acn3.52021 PMC11021617

[19] Velasco M, Zarco LA, Agudelo-Arrieta M, Torres-Camacho I, Garcia-Cifuentes E, Muñoz O. Effectiveness of treatments in Neuromyelitis optica to modify the course of disease in adult patients. Systematic review of literature. *Mult Scler Relat Disord*. 2021; 50: 102869. 10.1016/j.msard.2021.102869 33711580

[20] Akaishi T, Nakashima I, Takahashi T, Abe M, Ishii T, Aoki M. Neuromyelitis optica spectrum disorders with unevenly clustered attack occurrence. Neurol Neuroimmunol Neuroinflamm. 2020;7: e640. 31757816 10.1212/NXI.0000000000000640 PMC6935841

[21] Yamamura T, Araki M, Fujihara K, Okuno T, Misu T, Guo YC, et al. Exploring steroid tapering in patients with neuromyelitis optica spectrum disorder treated with satralizumab in SAkuraSky: A case series. Mult Scler Relat Disord. 2022;61: 103772. 35537314 10.1016/j.msard.2022.103772

[22] Nakamagoe K, Tanaka M, Igari K. Cases of aquaporin-4-positive neuromyelitis optica spectrum disorder with successful tapering of prednisolone to less than 3 mg/day after satralizumab administration. Neurol Sci. 2023;18:1–4. 10.1007/s10072-023-06754-4 PMC10024277 36933100

[23] Nakashima I, Nakahara J, Yasunaga H, Yamashita M, Nishijima N, Satomura A, et al. Real-world management of patients with neuromyelitis optica spectrum disorder using satralizumab: Results from a Japanese claims database. Mult Scler Relat Disord. 2024;84: 105502. 38401202 10.1016/j.msard.2024.105502

[24] Kleiter I, Traboulsee A, Palace J, Yamamura T, Fujihara K, Saiz A, et al. Long-term efficacy of satralizumab in AQP4-IgG–seropositive neuromyelitis optica spectrum disorder from SAkuraSky and SAkuraStar. Neurol Neuroimmunol Neuroinflamm. 2023;10: e200071. 36724181 10.1212/NXI.0000000000200071 PMC9756307

[25] Yamamura T, Weinshenker B, Yeaman MR, De Seze J, Patti F, Lobo P, et al. Long-term safety of satralizumab in neuromyelitis optica spectrum disorder (NMOSD) from SAkuraSky and SAkuraStar. Mult Scler Relat Disord. 2022;66: 104025. 36007339 10.1016/j.msard.2022.104025

[26] Akaishi T, Takahashi T, Fujihara K, Misu T, Abe M, Ishii T, et al. Risk factors of attacks in neuromyelitis optica spectrum disorders. J Neuroimmunol. 2020;15(343): 577236. 10.1016/j.jneuroim.2020.577236 32279020

[27] Wingerchuk DM, Fujihara K, Palace J, Berthele A, Levy M, Kim HJ, et al. Long-term safety and efficacy of eculizumab in aquaporin-4 IgG-positive NMOSD. Ann Neurol. 2021;89:1088–98. 33586143 10.1002/ana.26049 PMC8248139

[28] Rensel M, Zabeti A, Mealy MA, Cimbora D, She D, Drappa J, Katz E. Long-term efficacy and safety of inebilizumab in neuromyelitis optica spectrum disorder: Analysis of aquaporin-4–immunoglobulin G–seropositive participants taking inebilizumab for ≥4 years in the N-MOmentum trial. Mult Scler. 2022;28:925–32. 34595983 10.1177/13524585211047223 PMC9024030

[29] Wingerchuk DM, Zhang I, Kielhorn A, Royston M, Levy M, Fujihara K, et al. Network meta-analysis of food and drug administration-approved treatment options for adults with aquaporin-4 immunoglobulin G-positive neuromyelitis optica spectrum disorder. Neurol Ther. 2022;11:123–35. 34773597 10.1007/s40120-021-00295-8 PMC8857350

[30] Tahara M, Oeda T, Okada K, Kiriyama T, Ochi K, Maruyama H, et al. Safety and efficacy of rituximab in neuromyelitis optica spectrum disorders (RIN-1 study): a multicentre, randomised, double-blind, placebo-controlled trial. Lancet Neurol. 2020;19:298–306. 32199095 10.1016/S1474-4422(20)30066-1

[31] Tahara M, Oeda T, Okada K, Ochi K, Maruyama H, Fukaura H, et al. Compassionate open-label use of rituximab following a randomised clinical trial against neuromyelitis optica (RIN-2 study): B cell monitoring-based administration. Mult Scler Relat Disord. 2022;60: 103730. 35287025 10.1016/j.msard.2022.103730

[32] Pittock SJ, Barnett M, Bennett JL, Berthele A, de Sèze J, Levy M, et al. Ravulizumab in aquaporin-4-positive neuromyelitis optica spectrum disorder. Ann Neurol. 2023;93:1053–68. 36866852 10.1002/ana.26626

